# Effect of Plant Preservative Mixture^TM^ on Endophytic Bacteria Eradication from In Vitro-Grown Apple Shoots

**DOI:** 10.3390/plants11192624

**Published:** 2022-10-05

**Authors:** Natalya V. Romadanova, Arman B. Tolegen, Svetlana V. Kushnarenko, Elena V. Zholdybayeva, Jean Carlos Bettoni

**Affiliations:** 1 Institute of Plant Biology and Biotechnology, 45 Timiryazev St., Almaty 050040, Kazakhstan; 2 Department of Molecular Biology and Genetics, Al-Farabi Kazakh National University, Al-Farabi Av. 71, Almaty 050040, Kazakhstan; 3 National Center for Biotechnology, 13/5 Kurgalzhynskoye Road, Nur-Sultan 010000, Kazakhstan; 4 The New Zealand Institute for Plant and Food Research Limited, Batchelar Road, Palmerston North 4410, New Zealand

**Keywords:** *Bacillus megaterium*, biocide, *Malus*, microbial contamination, plant tissue culture, PPM^TM^

## Abstract

Endophytic contaminants are a common problem for the in vitro propagation of woody plants and have significant economic repercussions for the conservation of plant genetic resources and commercial micropropagation. In this study, first, the microbial contamination that appeared around the base of in vitro-grown apple shoots was identified as *Bacillus megaterium*. Then, plant preservative mixture (PPM^TM^) was used as a bactericidal agent in plant tissue culture. Its efficacy for eradicating endophytic *B. megaterium* in in vitro cultures of apple was tested. In vitro-contaminated shoots were grown in tissue culture medium supplemented with 0.2% *v*/*v* PPM^TM^ for 12 weeks and then transferred to medium without any PPM^TM^ and cultured for 24 weeks. This study showed that PPM^TM^ is an effective agent for controlling the growth of *B. megaterium*. Our results highlight the species-specific response of apple shoots to PPM^TM^. PPM^TM^ was effective in controlling endogenous microbial contaminations from apple varieties ‘Golden Delicious’, ‘Landsberger Renette’, ‘Suislepper’, and ‘Aport krovavo-krasnyi’; meanwhile, in ‘KG 7’ and ‘Gold Rush’, all the plants grown in the absence of PPM^TM^ were still bacterially contaminated, even though they were pre-treated for 12 weeks in PPM^TM^-supplemented medium. These results therefore suggest the essentiality of further testing of extended incubation of PPM^TM^ in these cultivars that had outbreaks of bacterial contamination.

## 1. Introduction

Micropropagation is one of the advanced methods for vegetative propagation of plants [1,2,3]. In vitro tissue culture supplements conventional propagation methods in the production of healthy, disease-free plants and allows the propagation of a large number of progeny plants with desirable traits throughout the year in a short time and reduced space [3,4,5,6,7,8]. In addition, micropropagation plays a key role in the conservation of plant genetic resources [1,9,10].

A micropropagation protocol proceeds through a series of stages, starting with the establishment of aseptic cultures [3]. Avoiding microbial contamination of plant tissue cultures is critical to successful micropropagation [11]. Despite extensive surface sterilization and the use of best practices for culture maintenance, the contamination of plant tissue may be a persistent problem during micropropagation [12,13,14]. This source of contamination is known as endophytes, which are of particular concern because they can survive surface sterilization [14]. Endophyte contamination causes culture losses and directly affects the efficiency of micropropagation protocols [13]. These microorganisms live within plant tissues and may not be visible until after several rounds of subculture and/or after the cultures have undergone some stress [12,13,14,15]. For example, many plant species have shown that bacterial infection may not always became apparent at the early initial stage of tissue culture; however, after an extended culture duration or any other stresses, such as a long period without subculture, bacterial haze can appear around the base of cultures that initially appeared to be clean and can therefore pose a significant problem for the propagation and distribution of in vitro plants. Therefore, the elimination of bacterial infections in plant tissue culture is essential for the safe in vitro conservation and exchange of germplasm [16].

Recently, endophytic bacteria have been studied extensively due to their synergic effects on the host plants, such as the promotion of plant growth, the production of secondary metabolites, and plant adaptation to stressful environments [17,18,19,20,21,22]. However, when it comes to plant tissue culture, endophytic bacteria have often been described as contaminants. Many studies have been carried out to identity the diversity of the endophytic bacterial community in micropropagated plants. The most frequently found genera of bacterial endophytes are *Pantoea*, *Curtobacterium*, *Bacillus, Corynebacterium, Mycobacterium*, *Rhodopseudomonas*, *Pseudomonas*, and *Microbacterium* [23,24,25]. Although usually treated as contaminants, there are examples in which endophytic bacteria appear to have a beneficial effect when introduced within tissue culture systems, increasing multiplication and rooting rates as well as improving the effectiveness of in vitro micropropagation [13,23,26,27,28].

In some cases, contamination by latent bacterial infection often does not visually affect the growth of the micropropagated plants; however, when shoot tips are excised from in vitro cultures and exposed to stressful conditions, such as cryopreservation, the process triggers outbreaks of endophytes [29,30,31]. Heavy bacterial colonization on the shoot tips compromises the ability of explants to regenerate, and they often die when overwhelmed by contamination [30,31,32,33]. Previously, broad-spectrum antibiotics, such as ampicillin, gentamicin, amoxiclav, etc., were used to suppress or eliminate endophytic pathogens [34,35,36]. However, side effects of phytotoxicity on the growth of explants or a lack of response of some cultivars to the treatments associated with the continued use of antibiotics can lead to resistant strains, which are common problems [34,36,37,38,39]. Therefore, judicious use of antibiotics is especially important, considering their use as a last resort and only for short-term applications [40,41,42]. In addition, bacteria can remain present latently in plant tissue and contamination may reappear months after antibiotics are removed from the cultures, which only demonstrates the suppressive effect of these substances on the bacterial endophytes [16,36,39,43]. 

The Plant Preservative Mixture (PPM^TM^) is an effective approach to controlling endogenous contamination in plant tissue cultures [44]. PPM^TM^ is a broad-spectrum biocide/fungicide for plant tissue culture comprising two isothiazolones, namely 5-chloro 2-methyl 3 (2H) isothiazolone and 2-methyl-3 (2H) isothiazolone (Patent No. 5,750,402). In addition, PPM^TM^ composition includes other salts such as magnesium chloride, magnesium nitrate, potassium sorbate, and sodium benzoateisothiazolones, which, together with a mixture of two isothiazolones, act by inhibiting specific fundamental processes in the Krebs cycle and the electron transport chain [45,46]. In the 1990s, the manufacturer of PPM^TM^, Plant Cell Technology (Washington, D.C., USA), introduced the microbicide to the market, and since then, it has been successfully applied to control a wide range of microbes in plant tissue cultures of many fruit crops, including citrus, grapevine, papaya, berries, and others [32,47,48,49,50]. PPM^TM^ has advantages over antibiotics: PPM^TM^ is heat-stable and thus can be autoclaved with culture media; moreover, mutations against PPM^TM^ are not impossible but unlikely, because PPM^TM^ targets and inhibits multiple enzymes of bacteria, and antibiotic resistance is a well-known single-gene event [42,44,46]. PPM^TM^ has been demonstrated as effective in preventing microbial contamination at the establishment of in vitro cultures and during the course of tissue culturing [29,43,47,49,50,51,52]. As shown in early studies, the efficacy of PPM^TM^ for eradicating endophytes varies depending on the plant species and endogenous microbial contaminants. Furthermore, as with any antimicrobial compound, PPM™ may have deleterious effects on the establishment and growth of tissue culture plants of certain species [45]; therefore, in addition to its potential for controlling endogenous contaminants, its impact on plant growth should be tested before using it at a commercial level. 

In view of the reported efficacy of PPM™ for controlling endogenous microbial contaminants, this study was taken up to assess the effectiveness of PPM^TM^ at the concentration recommended by its manufacturer in eradicating endophytes from contaminated, in vitro-grown apple shoots. Currently, there are no previously published studies on PPM^TM^ use to eradicate endophytes from in vitro-grown apple cultures.

## 2. Results and Discussion

Endophytic contaminants have been a common problem for the in vitro propagation of woody plants and have significant economic repercussions for the conservation of plant genetic resources and commercial micropropagation [14,53,54]. Although different strategies have already been described to minimize the deleterious effects of endophytes, the effectiveness of the treatment may be dependent on the plant species and cultivar, the source of materials, propagation methods, and explant tolerance [29,32]. Culture media containing antimicrobial compounds may suppress or eliminate microbial growth [32,42,43,44]. Although some success has been reported using antibiotics, they often suppress the growth of bacteria rather than eliminate them from in vitro cultures [55,56,57,58]. In this study, as an alternative to using antibiotics, PPM^TM^ was tested for controlling the endophyte contaminants present in in vitro cultures of apple. 

The microbial contaminants that appeared around the base of the in vitro-grown apple shoots and on the surface of the medium were identified as *Bacillus megaterium* de Bary. Strains identified by matrix-assisted laser desorption/ionization time-of-flight mass spectrometry (MALDI-TOF/MS) had a score varying from 1.7 to 2.2, indicating reliable identification (Supplementary Table S1). *B. megaterium* is a relatively harmless Gram-positive, rod-shaped bacterium found in diverse habitats but commonly in soil [59,60,61,62,63]. This bacterium has already been identified as an endophytic contaminant in several plant species such as cotton [64], sweet corn [64], coffee [65], ginger [66], black pepper [67], Ilex paraguariensis [68], etc. 

Previously, bacterial strains of the genera *Bacillus* were recognized as a safe group, producing substances that are beneficial for plant development [69,70,71]. *B. megaterium* isolates are known to promote plant growth [72,73,74,75] and biocontrol ability against plant pathogens [67,76,77,78,79,80]. Recently, Wang et al. [81] showed that *B. megaterium* (WL-3 strain) had a strong ability to control late blight (*Phytophthora infestans*), one of the most destructive diseases in potato. They observed the antagonistic effect of *B. megaterium* against potato late blight, as well as the promotion of potato plant growth in plant tissues in vitro [81]. Similarly, working on the mulberry tree, Ou et al. [82] found that *B. megaterium* (HGS7 strain) consistently exhibited antagonistic activity against ten phytopathogens in vitro, especially for *Sclerotinia sclerotiorum* and *Scleromitrula shiraiana*. In addition to the antifungal traits, they also found that *B. megaterium* had strong tolerance to abiotic stress both in vitro and in planta [82]. Several studies have shown that endophytes are common in tissue culture and demonstrated their beneficial effects as plant growth-promoting agents [13,23,26,27,28,83,84,85,86]. Interestingly, working on apple plants grown in vitro, Tamošiūnė et al. [87] noted contrasting effects of bacterization with endophytic bacteria strains of *Bacillus* and *Pseudomonas* spp. on shoot biomass accumulation and the proliferation of auxiliary shoots. They observed that the stimulating or suppressing effect on shoot growth and proliferation was related to the specific interaction between plant host and endophytic bacteria strain [87].

Herein, latent or non-latent infections by *B. megaterium* did not affect the growth of the apple shoot cultures; however, when shoot tips, apparently from clean in vitro plants, were exposed to the cryopreservation procedure, endophyte outbreaks arose and compromised the shoot tip regrowth (Supplementary Figure S1). The negative impact of endophytes on the recovery of the cryopreserved shoot tips was previously reported [30,31,32,33]. Recently, Bettoni et al. [49] found that the addition of 1.5% PPM^TM^ (*v*/*v*) to pretreatment and preculture media was effective at controlling endophytic bacteria in cryopreserved *Vitis* shoot tips. They observed that the addition of PPM^TM^ prior to shoot tip excision was essential to eliminate endogenous microbial contaminants during the recovery process [49]. Beneficial effects for *Vitis*, however, may not be evident if PPM^TM^ (1.5% *v*/*v*) is added only to the regrowth medium; despite still having a suppressive effect on the growth of contaminants, it has also been shown to have a negative impact on shoot tip regrowth (J.C. Bettoni, personal communication). Therefore, to effectively carry out future cryopreservation experiments, we are faced with the task of eliminating endogenous microbial contaminants by *B. megaterium* from in vitro apple plants. In vitro-contaminated shoots were grown in Murashige and Skoog [88] (MS)-based tissue culture medium with the incorporation of 0.2% *v*/*v* PPM^TM^ for two cycles of 6 weeks each (Experiment 1, E1) and then transferred to the same medium but without PPM^TM^ and cultured for an additional four cycles of 6 weeks each (Experiment 2, E2). It was possible to control the identified endophyte contaminants using PPM^TM^; however, the efficacy of the treatments varied according to the apple cultivar as well as the PPM^TM^ incubation period (Table 1).

**Figure 1:**
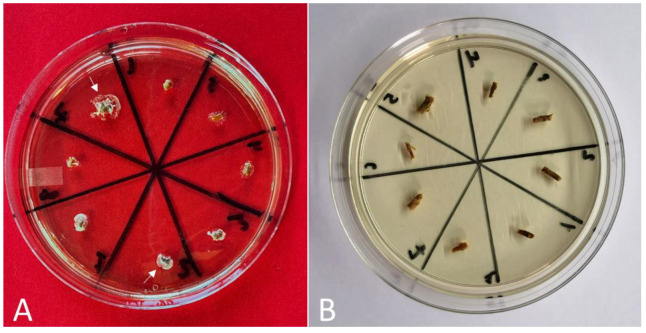
Indexing in vitro *Malus* spp. shoots for the presence of contamination on bacteriological growth medium 523. Shoots were grown 12 weeks (two subcultures of 6 weeks each) on medium supplemented with PPM^TM^ (0.2% *v*/*v*) and then transferred to medium without any PPM^TM^ and cultured for four additional subcultures (total of 24 weeks of culture). (**A**) ‘Gold Rush’. (**B**) ‘Golden Delicious’. White arrows in A indicate the bacterial growth on and around the ‘Gold Rush’ explants and on bacteriological growth medium 523.

The presence of bacterial colonies was assessed by placing pieces of the base of the shoots that were subjected to PPM^TM^ treatment in bacterial indexing medium 523 (Figure 1). This is a rich medium containing sucrose as the energy source, vitamins from yeast extract, and amino acids from hydrolyzed casein, so it would support the growth of many bacteria. Bacterial colonies were observed in 100% of the shoots derived from control plants (without PPM^TM^ treatment) after 3 weeks on medium 523, suggesting the effectiveness of this bacteriological medium in indexing *B. megaterium*. Notably, our results indicate that PPM^TM^ controlled *B. megaterium* in shoots cultured in PPM^TM^-supplemented medium (0.2% *v*/*v*) for two cycles of 6 weeks (E1) for all cultivars tested. Aseptic shoot levels ranging from 62.5% to 100% were obtained across six apple cultivars; on average, 79.2% of in vitro shoots in E1 were free of bacteria (Table 1, E1). Shoots derived from E1 were then transferred to medium without any PPM^TM^ and cultured for an additional four subculture cycles of 6 weeks each (total of 24 weeks) before being observed for bacterial growth (E2). After 24 weeks of culture, bacterial contamination was completely controlled in in vitro shoots of apple varieties ‘Golden Delicious’ and ‘Landsberger Renette’, while 62.5% of ‘Suislepper’ and 75% of ‘Aport krovavo-krasnyi’ shoots were found to be free of bacterium, suggesting that the bacterium was either suppressed to very low levels or eliminated. Interestingly, in contrast, we found that apple varieties ‘KG 7’ and ‘Gold Rush’ grown in the absence of PPM^TM^ had outbreaks of bacterial contamination around the base of the shoots and on the surface of the medium (Table 1, E2). Similarly, aiming to control bacterial contamination in in vitro cultures of switchgrass, Wang et al. [89] found that the growth of six endophytic bacterial strains was completely suppressed from cultures grown in a medium containing 0.2% PPM^TM^; however, the effectiveness of eradicating the bacteria was only achieved when PPM^TM^ was used throughout the micropropagation process; otherwise, in the absence of PPM^TM^, an outbreak of bacterial contamination was observed. Therefore, the addition of 0.2% PPM^TM^ to the growth media during the entire cultivation process may be a possible alternative to suppress the outbreak of bacterial contamination in ‘KG 7’ and ‘Gold Rush’ apple cultures.

Several studies have demonstrated the positive effect of PPM^TM^ to control endophyte contamination when it is used at the proper concentration, which was indicated to be different depending on the plant species [32,43,45,47,48,50,90,91,92,93]. Here, we used the PPM^TM^ manufacturer’s concentration instructions for controlling endogenous contamination in woody plants. The results obtained in the present study clearly demonstrate that the effectiveness of the proposed procedure for eradicating endophytic contamination was cultivar-specific. Therefore, future research is needed to access the effect of different incubation periods of in vitro shoots of apple ‘KG 7’ and ‘Gold Rush’ in growth medium supplemented with PPM^TM^ at different concentrations to control *B. megaterium* contamination. Furthermore, the effectiveness of PPM^TM^ has been described as dependent on the plant species tested and, mainly, which endophytic bacteria are present in the plant tissue [45,48,91]. 

Although the use of PPM^TM^ can be effective in controlling endophyte contamination, it also has been shown to have a negative impact on in vitro plant growth [45,50,92,93,94,95]. The tolerance levels and response to PPM^TM^ treatments vary greatly with species, type of plant tissue, exposure duration, and concentration, so preliminary testing is necessary for successful treatment, especially in sensitive materials [29,93,94,95]. While in chrysanthemum, the addition of 0.2% (*v*/*v*) PPM^TM^ to the growth medium completely inhibited shoot regeneration from leaf explants [95], in walnut, 21.7% of clean shoots were obtained after combined treatment of shaking explants at 5% (*v*/*v*) PPM^TM^ for 10 min followed by 0.2% (*v*/*v*) PPM^TM^ in the initiation medium, although the percentage of necrosis increased significantly to 60% compared to 10% in media without PPM^TM^ [29]. In this study, no phytotoxicity to the in vitro plants from the PPM^TM^-supplemented medium was observed. Shoots grown on medium supplemented with PPM^TM^ for 12 weeks were healthy and showed average multiplication rates of 3.9 across the six cultivars that were equivalent to 3.8 in control plants (Supplementary Table S2). The results from these experiments demonstrate that the addition of 0.2% (*v*/*v*) PPM^TM^ to the culture medium for 12 weeks is a viable alternative for reducing, and in some cases controlling, endogenous contaminants from shoot cultures of apple after in vitro establishment, without any negative impact on the growth of in vitro cultures. The determination of the suitable PPM™ concentration and incubation period is an essential requirement for those apple cultivars that showed outbreaks of bacterial contamination when shoots were transferred to medium without any PPM™.

## 3. Materials and Methods

### 3.1. Plant Material and Growth Conditions

In vitro-contaminated shoots of *Malus sieversii* (Ledeb.) M. Roem., ‘KG 7’, *Malus domestica* Borkh. ‘Aport krovavo-krasnyi’, ‘Golden Delicious’, ‘Gold Rush’, ‘Landsberger Renette’, and ‘Suislepper’ were originally obtained from the Institute of Plant Biology and Biotechnology (Almaty, Kazakhstan) and used as the objects of the study. In vitro plantlets were maintained and subcultured every 4 to 6 weeks in glass culture vessels (237 mL) (PhytoTechnology Laboratories^®^, Lenexa, KS, USA) with MS basal medium containing 30 g L^−1^ sucrose, 2.2 μM N6-benzylaminopurine (BAP), 0.04 μM indole-3-butyric acid (IBA), 1.75 g L^−1^ Gelrite™ (PhytoTechnology Laboratories^®^, Lenexa, KS, USA), and 4 g L^−1^ agar (PhytoTechnology Laboratories^®^, Lenexa, KS, USA) [1,5,96]. The medium pH was adjusted to 5.7 using NaOH or HCl prior to autoclaving at 121 °C for 20 min. All chemicals were supplied by SPF Mediland (Almaty, Kazakhstan), unless otherwise specified. Cultures were grown at 24 ± 1 °C under a photoperiod of 16 h light with a light intensity of 40 µM m^−2^ s^−1^ with two types of OPPLE tubular fluorescent lamps: YK 21RR 16/G 21W 6500K RGB and YK 21RL 16/G 21W 4000K RGB, supplied by ElectroComplex in Corporation (Almaty, Kazakhstan). 

### 3.2. Isolation, Cultivation, and Identification of Endophytic Bacteria from In Vitro Plants

Shoots were isolated from six-week-old cultures and used for the isolation of bacterial contaminants. Initially, apparent bacterial contamination was detected visually, collected, and aseptically streaked in Petri dishes (100 mm × 15 mm) containing bacteriological growth medium 523, a general media for the growth of bacteria composed of 8 g L^−1^ casein hydrolysate (Sigma-Aldrich, St. Louis, MO, USA), 10 g L^−1^ sucrose, 4 g L^−1^ yeast extract, 2 g L^−1^ monopotassium phosphate (KH_2_PO_4_), 0.15 g L^−1^ magnesium sulfate (MgSO_4_ 7H_2_O), and 6 g L^−1^ Gelrite™ at pH 6.9 [97]. Plates were incubated at room temperature. After 48 h of incubation, the developed colonies were purified by repeating isolation. Solitary colonies of contamination were collected using swabs and subsequent immersion into a tube containing Amies medium [98]. To obtain pure culture, all samples were cultivated on esculin–gelatin medium with incubation at 37 °C for 48 h under anaerobic conditions [99]. Thereafter, isolates were identified by MALDI-TOF/MS (Bruker Daltonik GmbH, Bremen, Germany), as described by Kozhakhmetova et al. [99]. MALDI-TOF/MS scores of ≥1.7 were accepted for species assignment, and scores below 1.7 were considered unreliable [99]. Further micropropagation experiments were carried out with indexed shoots.

### 3.3. Elimination of Contaminant Endophytic Bacteria Using PPM^TM^

**Figure 2:**
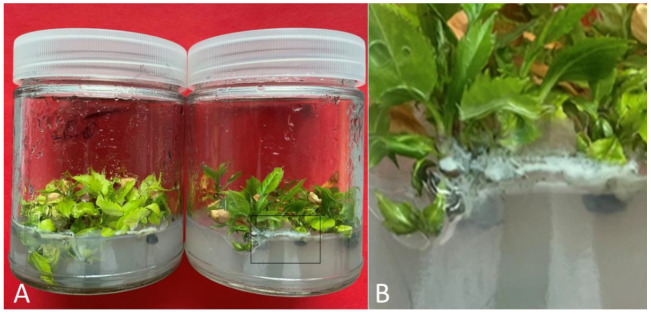
Apple (*Malus* spp.) in vitro cultures exhibiting microbial contamination. (**A**) Cultures exhibiting microbial contamination 6 weeks after subculture; (**B**) a closer view of cultures where bacterial colonies have formed around the base of shoots and on the surface of the medium.

Eight shoot segments (2 cm in length) for each cultivar were excised from six-week-old cultures with the presence of bacteria (Figure 2) and placed in individual test tubes (150 mm × 25 mm) with basal medium supplemented with 0.2% *v*/*v* PPM^TM^ (Plant Cell Technology, Washington, D.C., USA) and cultured in standard culture conditions. After 6 weeks of culture, shoot segments were transferred to fresh MS basal medium with 0.2% v/v PPM^TM^ and cultured for another 6 weeks. After 12 weeks of culture on medium with PPM^TM^ (two passages of 6 weeks each; E1), eight shoot segments for each cultivar were then transferred to basal medium without PPM^TM^ and cultured for four passages of 6 weeks each (total of 24 weeks of culture; E2). The control (C) comprised shoot segments excised from six-week-old cultures with the presence of bacteria and cultured on MS basal medium without PPM^TM^ for three passages of 6 weeks each (total of 18 weeks of culture). Shoot segments from E1, E2, and the control were then indexed for endophyte contamination using 523 detection medium [53]. For this, the basal parts of in vitro shoots (approx. 5 mm) were placed onto 100 × 15 mm Petri plates containing 25 mL of bacteriological growth medium 523 at a density of 8 shoots per plate. The Petri plates with shoots were incubated at 24 ± 1 °C under a photoperiod of 16 h light (40 µM m^−2^ s^−1^) for 2–3 weeks. In the absence of bacteria, the medium remains transparent, while the turbidity of the medium and the growth of colonies of microorganism near the base of the shoots indicate contamination.

### 3.4. Assessment of the Impact of PPM^TM^ on Shoot Multiplication

The multiplication rate (MR) was calculated in shoots grown for 12 weeks (two passages of 6 weeks each) in MS basal medium with 0.2% *v*/*v* PPM^TM^ and control without any PPM^TM^. Data were recorded during the subcultures. MR was determined by the formula: MR = a/bc, where a is the number of new shoots, b is the number of initial shoots, and c is the number of subcultures.

### 3.5. Statistical Analysis

Each experiment was performed with two replicates of eight shoots in each treatment, and the whole experiment was repeated twice. Data were expressed as the mean values ± standard error (SE). Least significant differences were calculated using one-directional ANOVA and Tukey’s mean separation test (*p* ≤ 0.05) using the software package SYSTAT 12.0 [100].

## 4. Conclusions

Although endophytic bacteria remain a continuing threat to plant tissue culture, techniques for controlling, and in some cases eliminating, contamination are available. In this study, application of the recommended concentration of PPM^TM^ (0.2% *v*/*v*) over 12 weeks of culture proved be effective to control the growth of *B. megaterium* in apple tissue cultures. Our results highlight the cultivar-specific response of apple shoots to PPM^TM^. These results therefore suggest the essentiality of further testing of extended incubation of PPM^TM^ in these cultivars that had outbreaks of bacterial contamination. It also provides a scope for extending PPM^TM^ application to other types of plants and endogenous contaminants. 

## Figures and Tables

**Table 1:** Percentage of aseptic shoots of in vitro cultures of apple (*Malus* spp.) grown for 12 weeks (two passages of 6 weeks each) in culture medium supplemented with 0.2% *v*/*v* Plant Preservative Mixture (PPM™) (Experiment 1, E1) and, subsequently, in pre-treated shoots from E1 grown in culture medium without PPM™ for 24 weeks (four passages of 6 weeks each) (Experiment 2, E2).

Species	Genotypes	Aseptic Shoots (%)								
		Control ^z^			Experiment 1 (E1)			Experiment 2 (E2)		
*M. sieversii*	‘KG 7’	0.0	±	0.0 ^a^	62.5 ^x^	±	3.6 ^b^	0.0	±	0.0 ^c^
*M. domestica*	‘Aport krovavo-krasnyi’	0.0	±	0.0 ^a^	87.5	±	2.9 ^b^	75.0	±	3.2 ^b^
*M. domestica*	‘Golden Delicious’	0.0	±	0.0 ^a^	100.0	±	0.0 ^a^	100.0	±	0.0 ^a^
*M. domestica*	‘Gold Rush’	0.0	±	0.0 ^a^	87.5	±	2.9 ^b^	0.0	±	0.0 ^c^
*M. domestica*	‘Landsberger Renette’	0.0	±	0.0 ^a^	75.0	±	3.2 ^b^	100.0	±	0.0 ^a^
*M. domestica*	‘Suislepper’	0.0	±	0.0 ^a^	62.5	±	3.6 ^b^	62.5	±	3.6 ^b^
Mean		0.0	±	0.0	79.2	±	3.8	56.3	±	6.7

^x^ Data represent mean ± standard error (SE). ^z^ Controls comprising shoot segments were excised from six-week-old cultures with the presence of bacteria and cultured on basal medium without PPM^TM^ for three passages of 6 weeks each (total of 18 weeks of culture). Values followed by different letters within each section were significantly different at *p* ≤ 0.05 using Tukey’s mean separation test.

## Data Availability

The datasets presented in the study are either included in the article or in the Supplementary Materials; further inquiries can be directed to the corresponding authors.

## Supplementary Materials

The following supporting information can be downloaded at: https://www.mdpi.com/article/10.3390/plants11192624/s1, Table S1: Result overview of identification of isolates by MALDI-TOF/MS; Table S2: Assessment of the impact of PPM^TM^ on shoot multiplication (multiplication rate = MR) of in vitro shoots of apple (*Malus* spp.) accessions grown in MS-based tissue culture medium with incorporation of 0.2% *v*/*v* PPM^TM^ for two cycles of 6 weeks each or without any PPM^TM^; Figure S1: Apple (*Malus* spp.) in vitro cultures exhibiting microbial contamination. Shoot tips exhibiting contamination 45 days after cryopreservation; bacterial colony growth was developed around shoot tips and on the surface of the medium during the recovery process.
