# Supplementary material for: Effect of Plant Preservative MixtureTM on Endophytic Bacteria Eradication from In Vitro-Grown Apple Shoots
Source: Plants (Basel). 2022 Oct 5;11(19):2624. doi: 10.3390/plants11192624 (PMC9572907; doi:10.3390/plants11192624)
Supplement: Supplementary file 1 [file plants-11-02624-s001.zip › plants-1943898-supplementary.pdf]

plants MDPI

Supplementary Material

Effect of Plant Preservative Mixture™ on endophytic bacteria eradication from in vitro-grown apple shoots

Supplementary Material

Table S1. Result overview of identification of isolates by matrix-assisted laser desorption/ionization time-of-flight mass spectrometry (MALDI-TOF/MS).

Analyte Name	Organism (best match)	Score Value	Organism (Second best match)	Score Value
C8	*Bacillus megaterium*	1.748*	unreliable identification	1.652
C9	*B. megaterium*	2.193	*B. megaterium*	2.085
D1	no peaks found	< 0	no peaks found	< 0
D10	*B. megaterium*	2.007	*B. megaterium*	1.888
D12	*B. megaterium*	2.124	*B. megaterium*	1.969

*Scores of ≥1.7 (highlighted in green) were accepted for species assignment and scores below 1.7 (highlighted in red) were considered unreliable [99].

Table S2. Assessment of the impact of plant preservative mixture (PPM™) on shoot multiplication rate (multiplication rate = MR) of *in vitro* shoots of apple (*Malus* spp.) accessions grown in Murashige and Skoog [88] (MS)-based tissue culture medium with incorporation of 0.2% v/v PPM™ for two cycle of six weeks each or without any PPM™.

Species	Genotypes	Control (without any PPM™)				0.2% v/v PPM™ for 12 weeks			
		Multiplication rate				Multiplication rate			
M. sieversii	'KG 7'	4.3 ^{z}	±	0.1	a	4.4	±	0.2	a
M. domestica	'Aport krovavo-krasnyi'	3.1	±	0.1	a	3.3	±	0.1	a
M. domestica	'Golden Delicious'	3.8	±	0.2	a	3.8	±	0.1	a
M. domestica	'Gold Rush'	3.5	±	0.1	a	3.3	±	0.1	a
M. domestica	'Landsberger Renette'	4.0	±	0.1	a	4.4	±	0.2	a
M. domestica	'Suislepper'	4.1	±	0.2	a	4.1	±	0.1	a
Mean		3.8	±	0.4		3.9	±	0.5	

* ^{z} Data represent mean ± standard error (SE). Values followed by different letters within each section were significantly different at p ≤ 0.05 using Tukey's mean separation test.

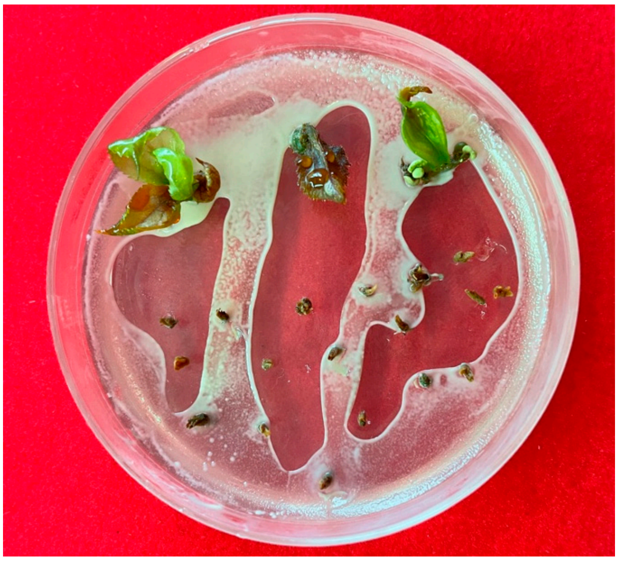

Figure S1. Apple (*Malus* spp.) *in vitro* cultures exhibiting microbial contamination. Shoot tips exhibiting contamination 45 days after cryopreservation; bacterial colony growth was developed around shoot tips and on the surface of the medium during the recovery process.
